# Supplementary material for: Successive DNA extractions improve characterization of soil microbial communities
Source: PeerJ. 2017 Feb 1;5:e2915. doi: 10.7717/peerj.2915 (PMC5291099; doi:10.7717/peerj.2915)
Supplement: Supplemental Information 1 [file peerj-05-2915-s001.pdf]

Successive DNA extractions improve characterization of soil microbial communities

Mauricio R. Dimitrov[1,2*], Annelies J. Veraart[2], Mattias de Hollander[2], Hauke Smidt[1], Johannes A. van Veen[2], Eiko E. Kuramae[2]

[1] Laboratory of Microbiology, Wageningen University, Wageningen, the Netherlands.
[2] Department of Microbial Ecology, Netherlands Institute of Ecology NIOO-KNAW, Wageningen, the Netherlands.

[*] **Corresponding author**
Mauricio R. Dimitrov
Phone: +31 317 473 496
Email: m.dimitrov@nioo.knaw.nl
Postal address: the Netherlands Institute of Ecology (NIOO-KNAW), Droevendaalsesteeg 10, 6708 PB, the Netherlands.

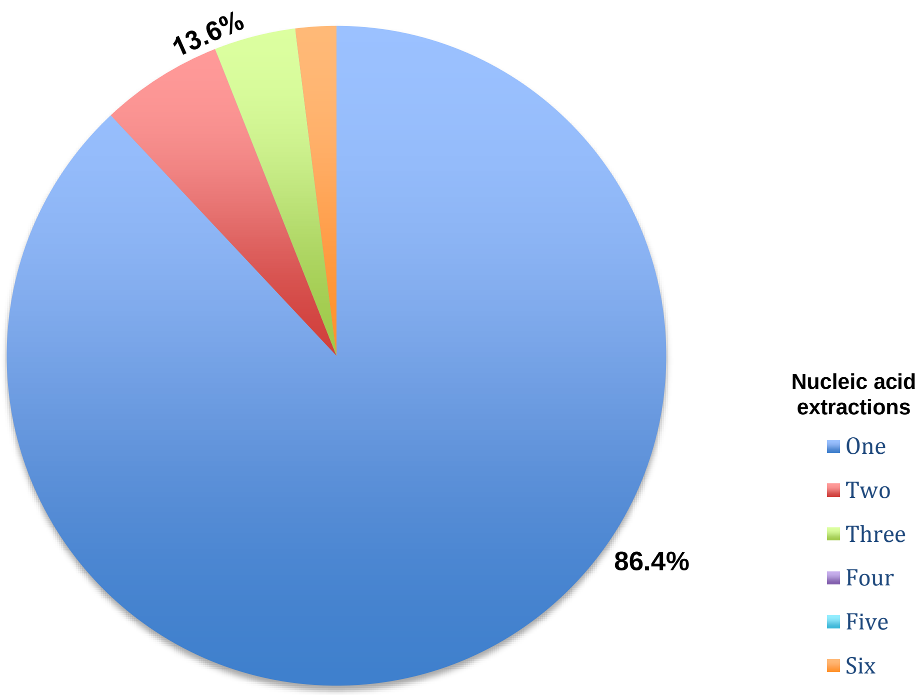

Figure S1. Analysis of fifty studies published in 2016 where one or multiple nucleic acid extractions were used to analyse soil microbial communities. Approximately 14% of the studies performed multiple acid nucleic extraction of the same soil sample. Data obtained by Web of Science search using topics: ‘soil AND microb*’ and ‘DNA OR RNA OR PCR OR metagen* OR "16S" OR sequencing’. Search was refined by using the following Web of Science categories: ‘SOIL SCIENCE’ AND ‘PUBLICATION YEARS’ (2016). The chart represents the first fifty obtainable studies in alphabetical order.

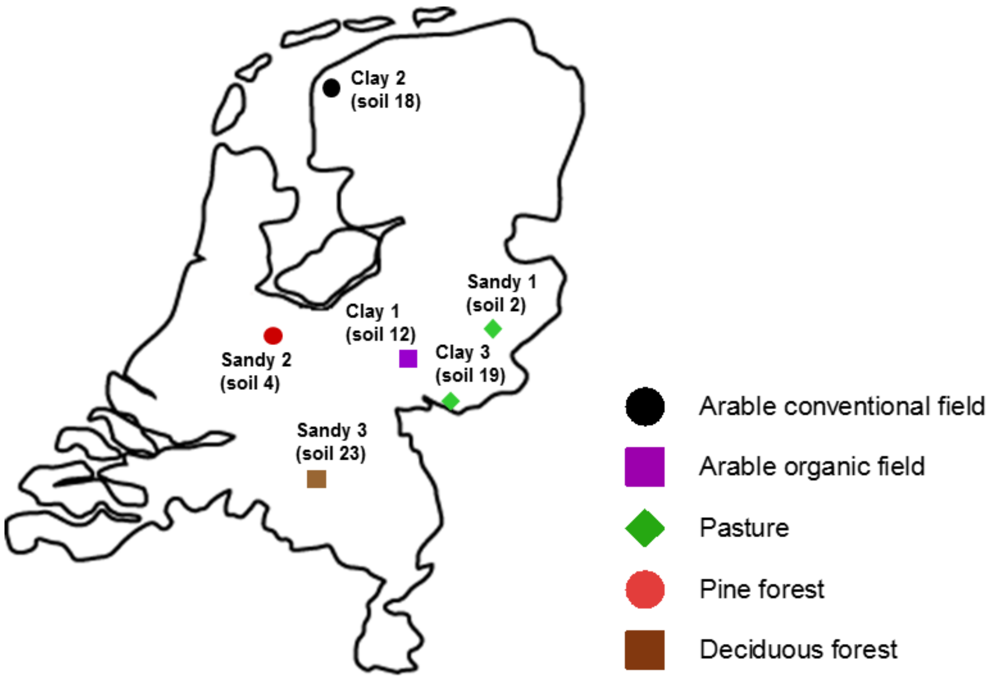

Figure S2. Overview of sampling locations and land management types of the soils used in the present work. For simplicity, samples have been renamed here. The identification, as found in Kuramae et al. (2012), is indicated between parentheses.

Table S1. Soil physical and chemical charactherists of the samples used in the present work.

Field	Land use	Latitude (N)	Longitude (E)	pH	Total N (%)	Total C (%)	C:N ratio	OM (%)	Total P (mg P_2O_5 $100g^{-1}$)	Clay (%)	Silt (%)	Sand (%)	$CaCO_3$ (%)	Cd (mg kg^{-1})	Cr (mg kg^{-1})	Cu (mg kg^{-1})	Ni (mg kg^{-1})	Pb (mg kg^{-1})	Zn (mg kg^{-1})	As (mg kg^{-1})	Hg (mg kg^{-1})
sandy soils																					
Sandy 1	Pasture	52º14"	06º41"	6.1	0.25	2.3	9.2	4	197	1.1	6.8	91.9	0	0.17	6.9	13	0	9.7	28	2.2	0
Sandy 2	Pine forest	52º08"	05º11"	3.7	0.17	3.8	22.3	6.4	17	0.3	4.3	95.4	0	0.1	0	0	0	15	0	1.8	0.04
Sandy 3	Deciduous forest	51º32"	05º18"	3.7	0.28	5	17.8	9	49	4.3	9.8	85.9	0	0.13	8.2	0	3.8	23	12	5	0.09
clay soils																					
Clay 1	Organic arable field	52º01"	06º12"	6.5	0.13	1.4	11.0	2.7	296	13.4	29.4	57.4	0.1	0.21	19	15	8.4	21	71	10	0.09
Clay 2	Conventional arable field	53º12"	05º31"	7.4	0.13	1.2	9.3	1.6	106	16.1	37.9	46	2.4	0.12	29	21	14	14	41	11	0.03
Clay 3	Pasture	51º53"	06º17"	6	0.55	4.8	8.7	9.4	310	36.7	51.1	12.1	0.2	0.49	52	27	33	32	130	14	0.08

Table S2. DNA yield (µg of DNA/g of soil) (SD) in successive extractions (E1-E3) for both kits in clay soils (A) and sandy soils (B). DNA quantification was performed using a Qubit 2.0 fluorometer.

A

	clay 1		clay 2		clay 3	
**Extraction**	**PS**	**FS**	**PS**	**FS**	**PS**	**FS**
E1	5.74(0.24)	21.35(14.07)	2.42(0.18)	8.13(0.71)	14.61(8.71)	42.57(8.47)
E2	3.05(0.25)	9.90(4.18)	3.18(0.52)	4.67(3.15)	0.33(0.29)	28.49(2.75)
E3	0.72(0.58)	5.00(0.43)	2.03(0.17)	6.95(1.28)	0.34(0.28)	20.97(17.09)

B

	sandy 1		sandy 2		sandy 3	
**Extraction**	**PS**	**FS**	**PS**	**FS**	**PS**	**FS**
E1	11.53(1.01)	31.90(8.36)	3.64(0.34)	10.68(2.62)	3.43(0.81)	12.00(8.68)
E2	4.67(1.22)	18.15(0.11)	1.90(0.04)	5.02(1.41)	3.81(0.77)	22.17(15.45)
E3	3.20(0.12)	8.62(0.49)	1.09(0.22)	3.07(0.60)	1.94(0.09)	5.12(1.29)

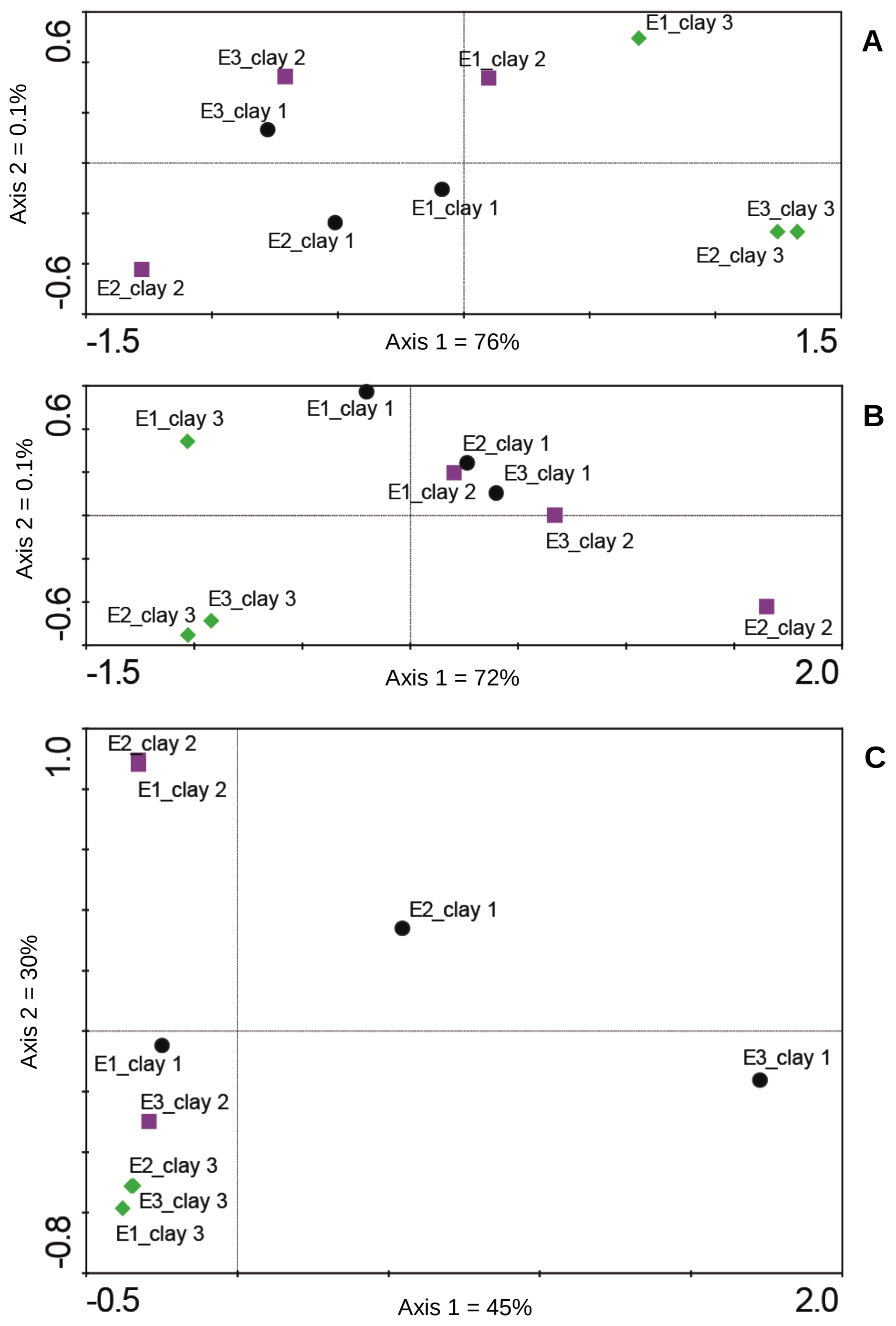

A
Axis 2 = 0.1%
0.6
-0.6
-1.5
1.5
Axis 1 = 76%
E1_clay 3
E3_clay 2
E1_clay 2
E3_clay 1
E1_clay 1
E2_clay 1
E3_clay 3
E2_clay 3
E2_clay 2
B
Axis 2 = 0.1%
0.6
-0.6
-1.5
2.0
Axis 1 = 72%
E1_clay 3
E1_clay 1
E2_clay 1
E3_clay 1
E1_clay 2
E3_clay 2
E3_clay 3
E2_clay 3
E2_clay 2
C
Axis 2 = 30%
1.0
-0.8
-0.5
2.0
Axis 1 = 45%
E2_clay 2
E1_clay 2
E2_clay 1
E1_clay 1
E3_clay 1
E3_clay 2
E2_clay 3
E3_clay 3
E1_clay 3

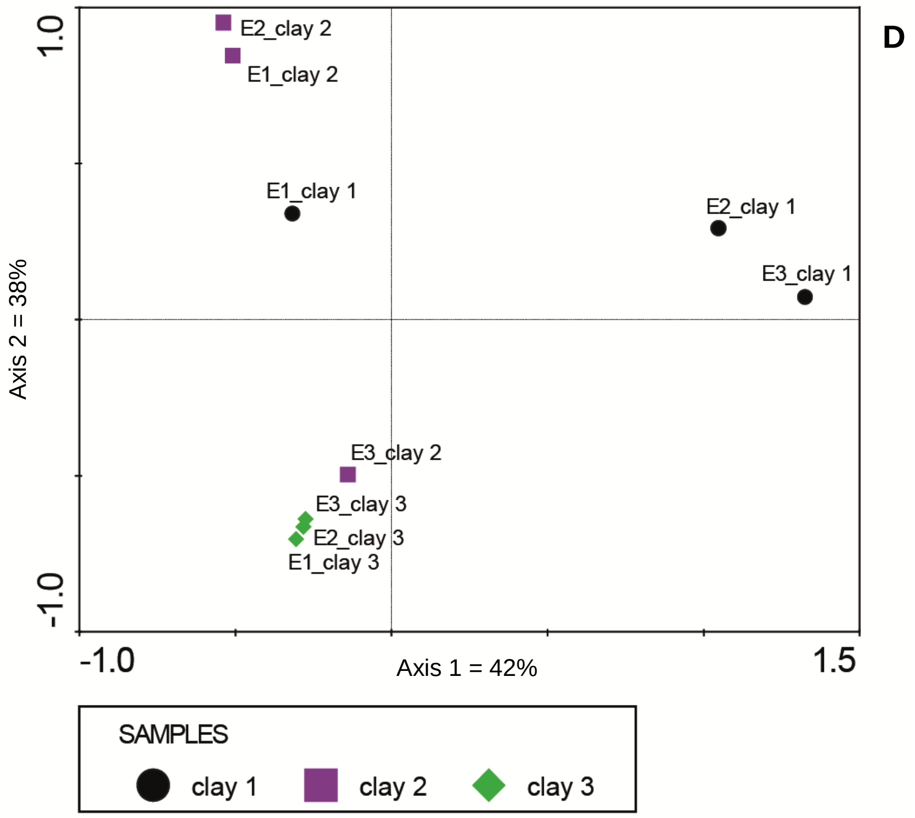

Figure S3. PCA plots of fungal community in clay soils analysed by T-RFLP. (A) total DNA extracted with PS and analysed using the forward primer; (B) total DNA extracted with PS and analysed using the reverse primer; (C) total DNA extracted with FS and analysed using the forward primer; (D) total DNA extracted with FS and analysed using the reverse primer.

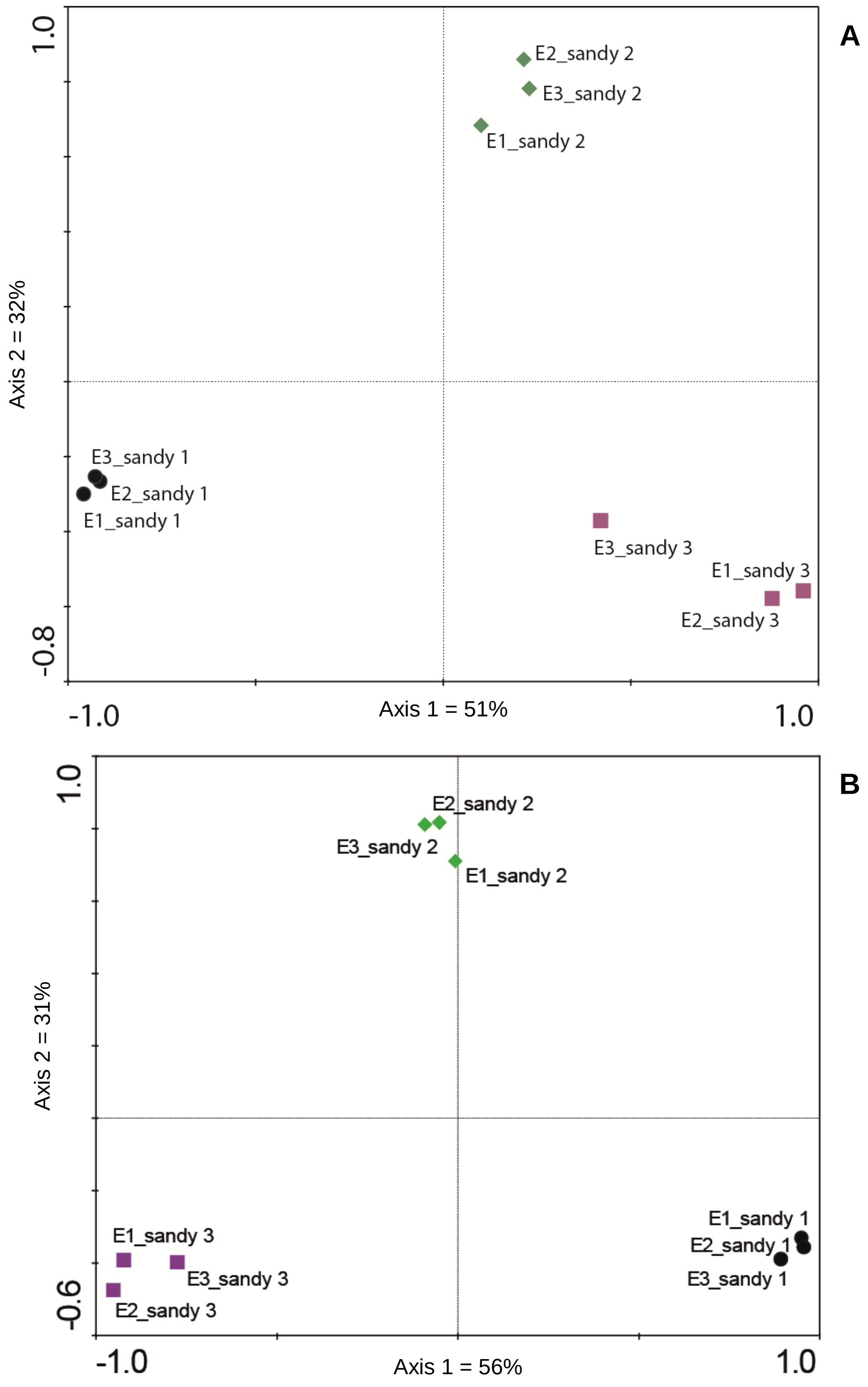

A
1.0
-0.8
Axis 2 = 32%
E2_sandy 2
E3_sandy 2
E1_sandy 2
E3_sandy 1
E2_sandy 1
E1_sandy 1
E3_sandy 3
E1_sandy 3
E2_sandy 3
-1.0
Axis 1 = 51%
1.0
B
1.0
-0.6
Axis 2 = 31%
E2_sandy 2
E3_sandy 2
E1_sandy 2
E1_sandy 3
E3_sandy 3
E2_sandy 3
E1_sandy 1
E2_sandy 1
E3_sandy 1
-1.0
Axis 1 = 56%
1.0

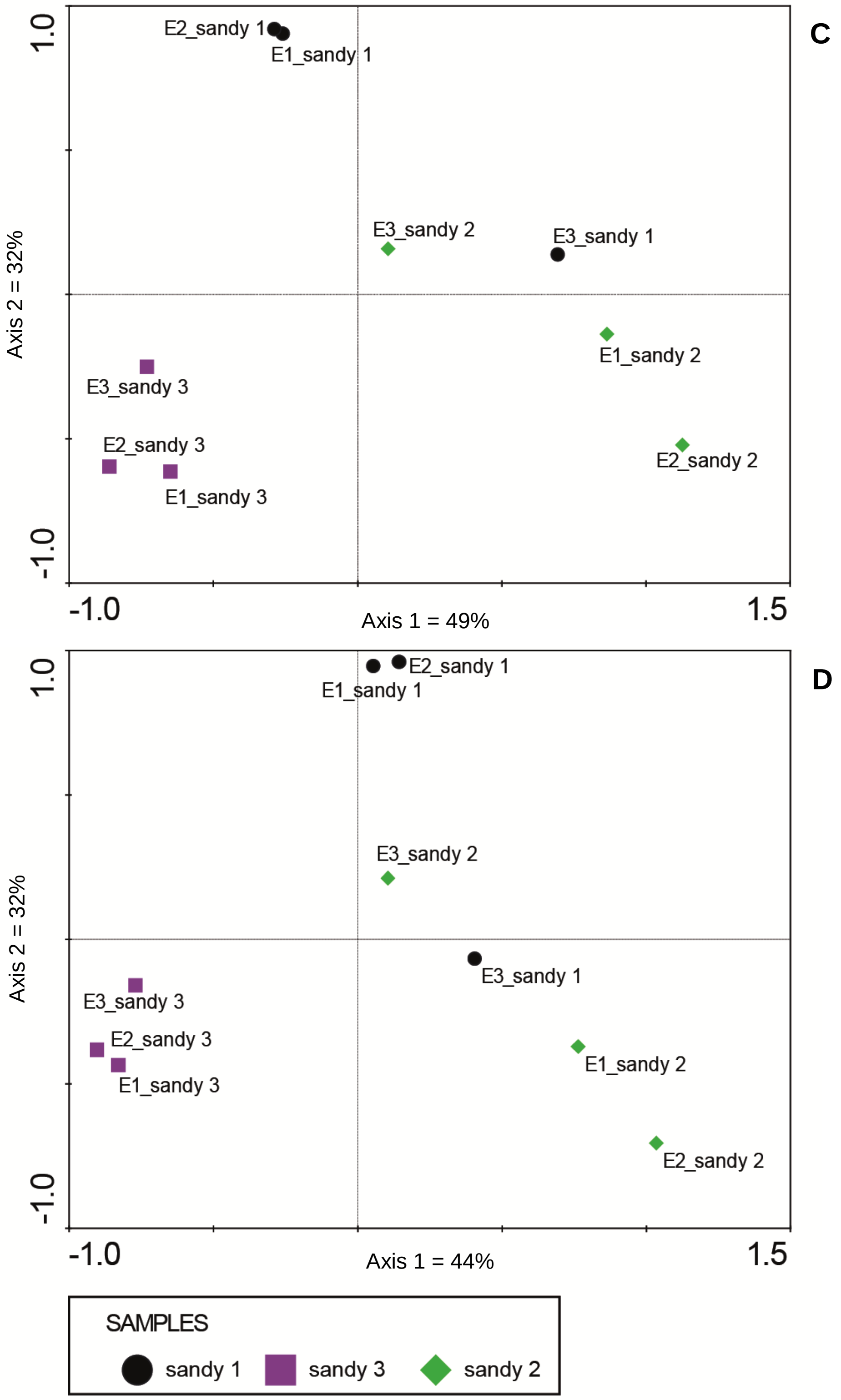

Figure S4. PCA plots of fungal community in sandy soils analysed by T-RFLP. (A) total DNA extracted with PS and analysed using the forward primer; (B) total DNA extracted with PS and analysed using the reverse primer; (C) total DNA extracted with FS and analysed using the forward primer; (D) total DNA extracted with FS and analysed using the reverse primer.

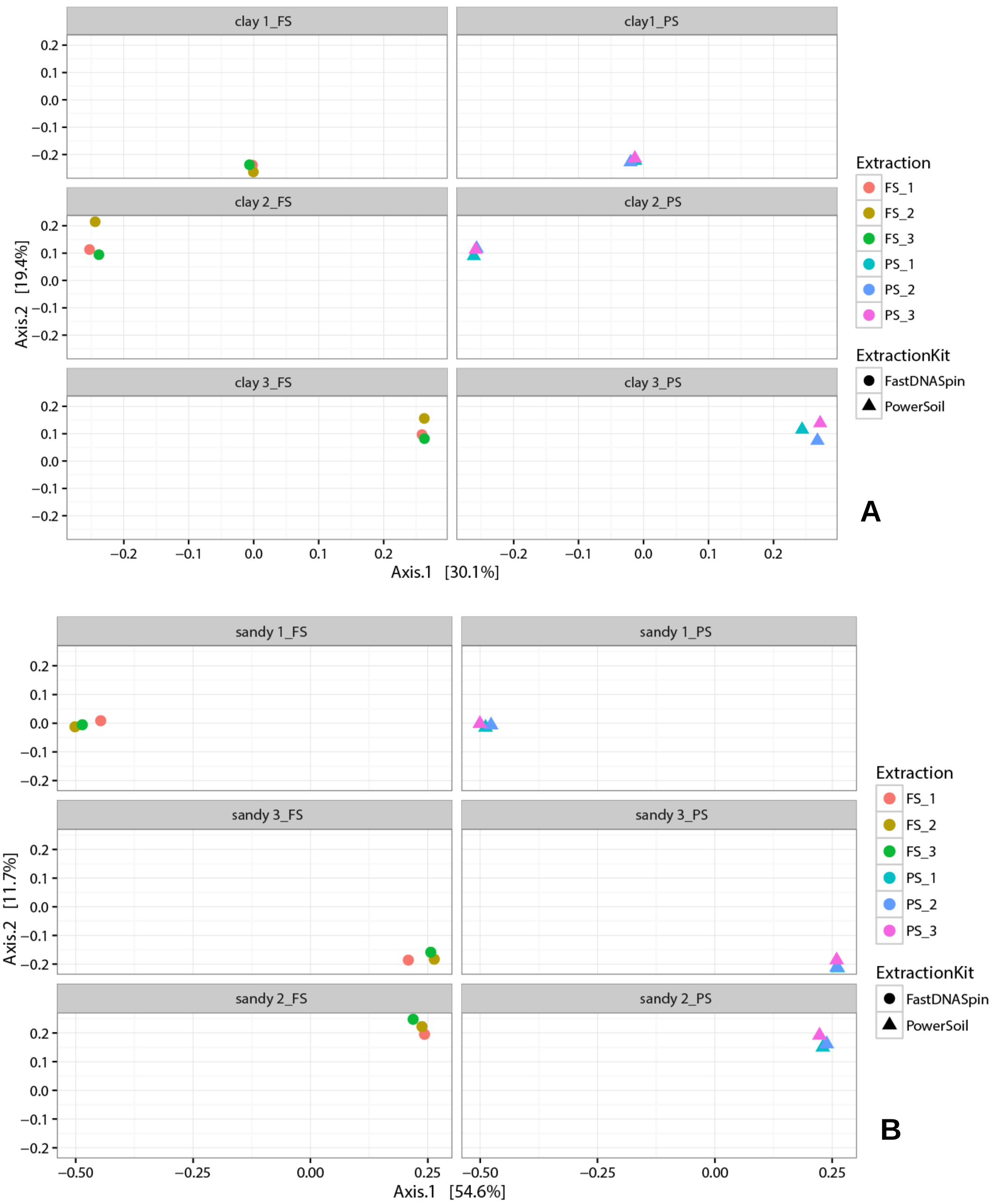

Figure S5. Multidimensional scaling (MDS) analysis of unweighted Unifrac values from prokaryotic community in clay (A) and sandy soils (B).

Table S3. Relative abundance of the ten most abundant bacterial Phyla in all clay (A) and sandy (B) soils.

A

Taxa	PS									FS								
	E1	E2	E3	E1	E2	E3	E1	E2	E3	E1	E2	E3	E1	E2	E3	E1	E2	E3
	clay1	clay1	clay1	clay2	clay2	clay2	clay3	clay3	clay3	clay1	clay1	clay1	clay2	clay2	clay2	clay3	clay3	clay3
Bacteria; Proteobacteria	28.93	17.92	24.58	22.19	18.06	16.75	17.39	18.70	17.66	24.78	24.04	21.85	16.59	9.46	14.67	11.22	10.05	10.49
Bacteria; Actinobacteria	14.09	10.53	19.40	12.74	11.22	13.38	27.17	19.27	20.16	14.62	13.97	15.16	12.48	9.27	15.09	15.58	10.18	10.45
Bacteria; Acidobacteria	12.06	14.88	13.42	17.11	18.96	15.97	10.30	15.01	10.79	14.34	11.69	8.76	21.28	24.24	16.00	11.41	10.14	5.85
Bacteria; Verrucomicrobia	2.59	4.43	3.43	4.50	3.18	2.56	18.91	20.62	26.22	2.95	3.59	3.57	3.35	6.38	4.02	32.72	45.74	50.25
Bacteria; Firmicutes	4.78	5.01	5.80	1.71	2.13	3.02	0.21	0.90	2.36	7.32	14.87	22.29	4.06	3.40	9.81	2.33	2.80	7.47
Bacteria; Chloroflexi	4.80	8.76	8.99	7.60	8.78	11.62	8.41	6.61	6.57	4.98	5.46	4.77	9.72	17.82	10.72	6.72	6.04	3.17
Bacteria; Planctomycetes	4.04	7.03	9.90	8.14	6.34	7.48	6.03	5.64	6.11	3.99	4.34	5.19	6.31	8.80	6.51	5.15	4.50	2.13
Archaea; Thaumarchaeota	3.16	16.32	2.37	6.87	13.12	15.28	0.18	0.76	1.88	4.16	6.32	4.17	7.16	5.72	10.22	3.85	2.93	4.83
Bacteria; Nitrospirae	1.58	1.92	2.11	2.08	2.58	2.19	2.56	5.99	3.40	2.40	1.70	1.01	2.99	5.88	2.14	3.92	3.36	1.08
Bacteria; Gemmatimonadetes	5.39	1.92	2.87	4.52	3.41	2.11	1.61	1.27	0.84	3.46	1.95	1.52	3.43	1.79	1.04	0.96	0.44	0.44

B

Taxa	PS									FS								
	E1	E2	E3	E1	E2	E3	E1	E2	E3	E1	E2	E3	E1	E2	E3	E1	E2	E3
	sandy1	sandy1	sandy1	sandy2	sandy2	sandy2	sandy3	sandy3	sandy3	sandy1	sandy1	sandy1	sandy2	sandy2	sandy2	sandy3	sandy3	sandy3
Bacteria; Proteobacteria	25.16	23.19	15.59	27.16	27.76	31.86	27.32	32.87	36.81	17.22	13.37	13.02	24.10	22.29	21.73	30.91	26.69	28.43
Bacteria; Actinobacteria	23.69	18.21	13.35	23.40	17.51	26.17	7.07	12.33	13.72	19.41	15.89	18.99	24.06	27.51	33.33	12.52	14.63	22.08
Bacteria; Acidobacteria	6.67	8.62	5.82	32.28	32.16	21.93	41.28	31.30	22.62	9.08	10.26	8.84	22.76	20.45	15.15	24.94	15.59	8.87
Bacteria; Verrucomicrobia	6.42	4.14	4.57	1.46	1.85	0.75	3.64	2.61	3.52	9.16	15.93	11.10	2.85	1.48	0.88	5.47	4.38	1.52
Bacteria; Firmicutes	15.24	28.27	44.33	0.15	0.45	0.56	0.11	0.34	0.82	14.41	17.79	20.24	0.19	0.40	1.07	0.59	0.89	1.80
Bacteria; Chloroflexi	4.60	3.20	2.87	1.73	2.99	3.05	1.83	2.31	5.28	8.14	9.20	10.70	4.31	4.63	5.68	5.00	10.26	11.56
Bacteria; Planctomycetes	3.36	2.75	3.08	2.56	6.66	4.83	2.06	2.82	5.79	4.80	5.71	8.19	7.36	7.33	8.41	3.95	8.85	9.60
Archaea; Thaumarchaeota	1.86	1.32	1.98	0.38	0.55	0.59	4.54	2.62	1.35	5.92	3.91	3.72	1.43	1.37	0.97	7.06	4.10	2.61
Bacteria; Nitrospirae	0.58	0.72	0.26	0.15	0.18	0.06	0.11	0.14	0.10	0.33	0.18	0.26	0.19	0.02	0.05	0.26	0.45	0.02
Bacteria; Gemmatimonadetes	2.09	1.73	0.93	0.24	0.24	0.29	0.18	0.12	0.10	1.36	0.87	0.97	0.15	0.11	0.12	0.24	0.16	0.08

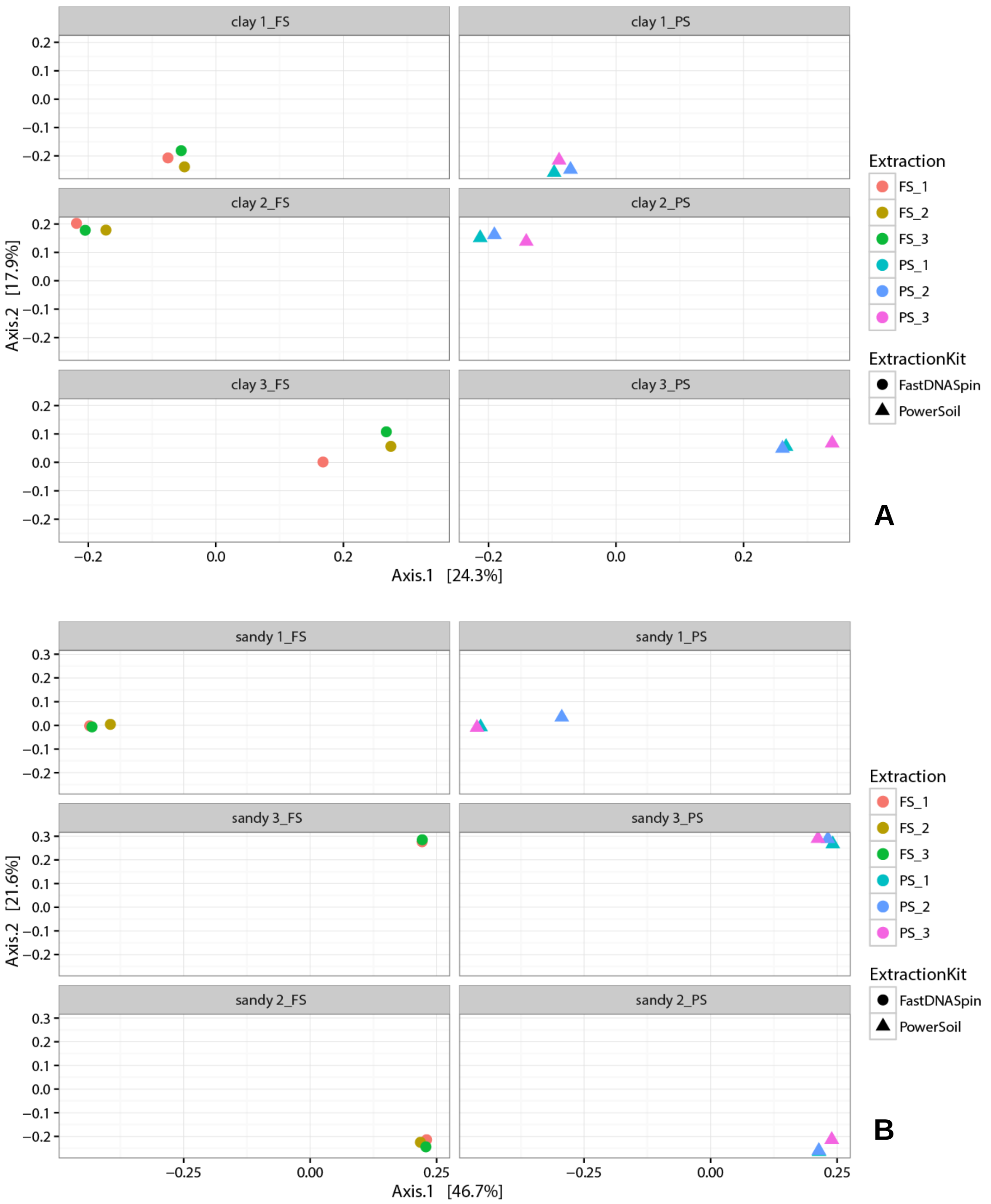

Figure S6. Multidimensional scaling (MDS) analysis of unweighted Unifrac values from fungal community in clay (A) and sandy soils (B).

Table S4. Archaeal phylum (A), bacterial phyla (B) and fungal phyla (C) observed only in E2 and/or E3.

A

Phylum	Soil sample
Euryarchaeota	clay 1 (PS) and clay 3 (FS)

B

Phylum	Soil sample
BD1-5	sandy 1 (FS)
BHI80-139	sandy 2 (FS)
Candidate_division_TM7	sandy 1 (FS)
Chlorobi	clay 3 (PS)
Cyanobacteria	clay 3 (PS)
Deinococcus-Thermus	clay 1 (PS) and sandy 1 (FS)
Elusimicrobia	clay 3 (PS)
Fibrobacteres	clay 2 (FS)
Lentisphaerae	clay 1 (FS), clay 3 (FS) and sandy 1 (FS)
SHA-109	clay 2 (PS)
SM2F11	clay 2 (PS) and clay 1 (FS)
TM6	clay 2 (PS)
Tenericutes	clay 1 (PS)
Thermotogae	sandy 2 (PS)
WCHB1-60	clay 1 (PS) and clay 3 (FS)
WD272	clay 1 (PS)

C

Phylum	Soil sample
Glomeromycota	sandy 1 (FS)
Other	clay 2 (FS) and sandy 2 (FS)
Unclassified	clay 2 (FS)

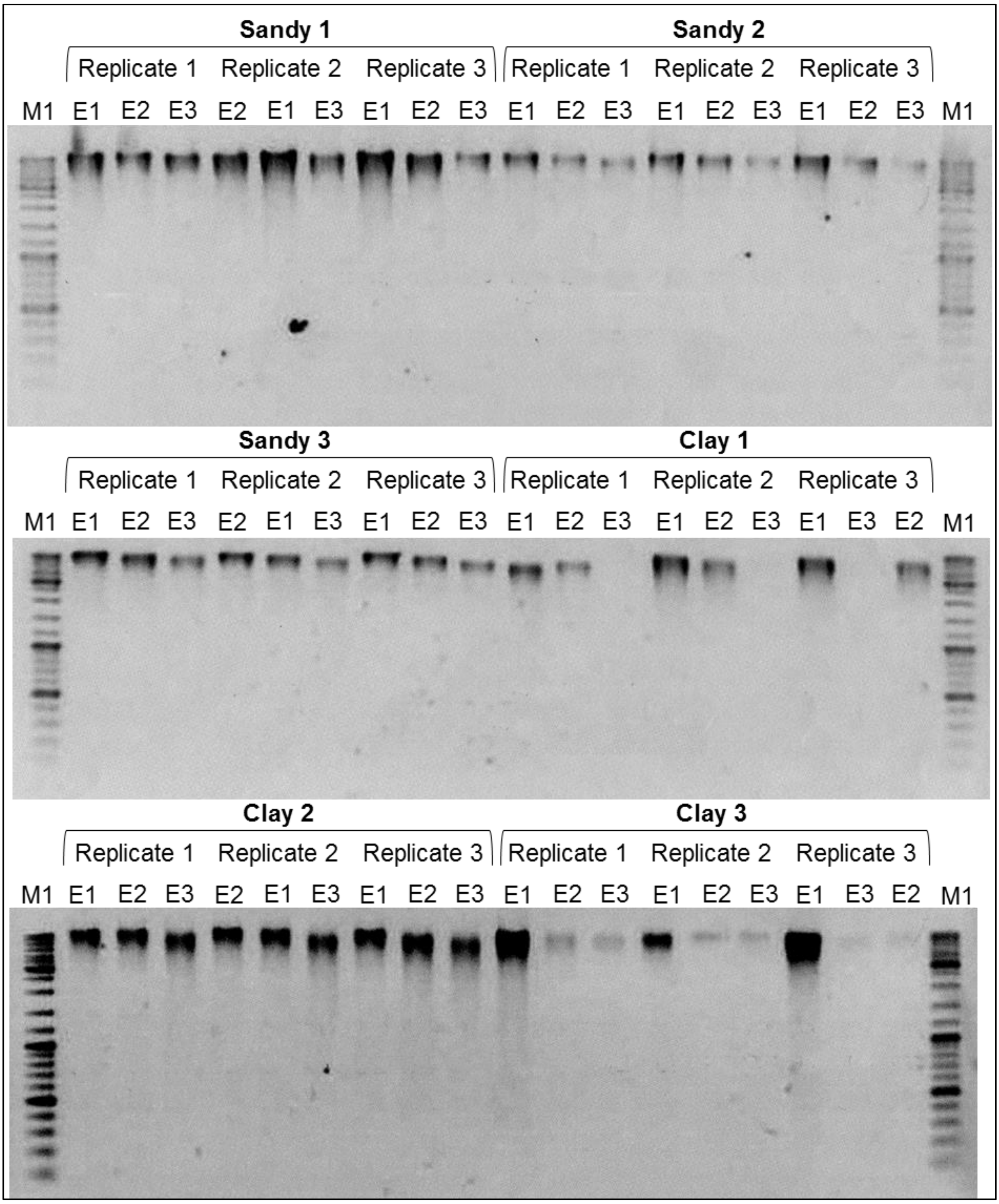
Sandy 1
Sandy 2
Replicate 1 Replicate 2 Replicate 3 Replicate 1 Replicate 2 Replicate 3
M1 E1 E2 E3 E2 E1 E3 E1 E2 E3 E1 E2 E3 E1 E2 E3 E1 E2 E3 M1
Sandy 3
Clay 1
Replicate 1 Replicate 2 Replicate 3 Replicate 1 Replicate 2 Replicate 3
M1 E1 E2 E3 E2 E1 E3 E1 E2 E3 E1 E2 E3 E1 E2 E3 E1 E3 E2 M1
Clay 2
Clay 3
Replicate 1 Replicate 2 Replicate 3 Replicate 1 Replicate 2 Replicate 3
M1 E1 E2 E3 E2 E1 E3 E1 E2 E3 E1 E2 E3 E1 E2 E3 E1 E3 E2 M1

A

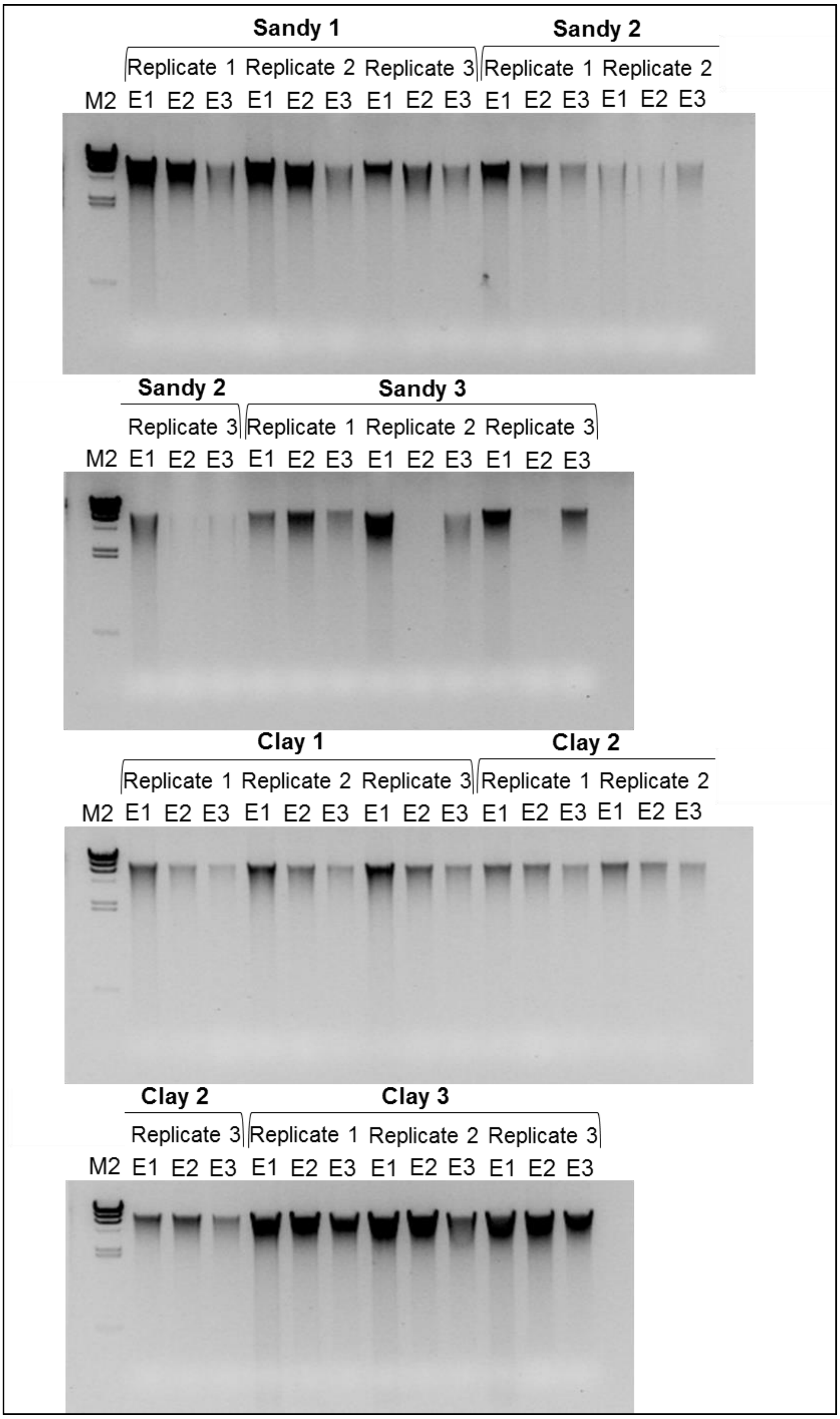

B

Figure S7. Visualization on agarose gel of the soil DNA extracted with PS (A) and FS (B). E = extraction, M1 = O'GeneRuler DNA Ladder Mix (Fermentas) and M2 = Lambda DNA/HindIII (Fermentas).

Table S5. Quality measurement (260/280 and 260/230 ratios) of DNA extracted with PS (A) and FS (B). Measurements were performed on NanoDrop 1000 spectrophotometer.

A

Clay			Sandy		
**Samples**	**260/280**	**260/230**	**Samples**	**260/280**	**260/230**
E1_clay_1_PS_R1	1.76	1.19	E1_sandy_1_PS_R1	1.84	1.57
E2_clay_1_PS_R1	1.52	0.89	E2_sandy_1_PS_R1	1.72	1.36
E3_clay_1_PS_R1	1.23	0.50	E3_sandy_1_PS_R1	1.62	0.96
E1_clay_1_PS_R2	1.78	1.22	E1_sandy_1_PS_R2	1.87	1.70
E2_clay_1_PS_R2	1.69	1.09	E2_sandy_1_PS_R2	1.79	1.38
E3_clay_1_PS_R2	1.63	1.10	E3_sandy_1_PS_R2	1.63	1.10
E1_clay_1_PS_R3	1.81	1.04	E1_sandy_1_PS_R3	1.85	1.47
E2_clay_1_PS_R3	1.76	0.99	E2_sandy_1_PS_R3	1.83	1.50
E3_clay_1_PS_R3	1.37	0.32	E3_sandy_1_PS_R3	1.63	0.97
E1_clay_2_PS_R1	1.71	0.76	E1_sandy_2_PS_R1	1.73	1.16
E2_clay_2_PS_R1	1.73	0.77	E2_sandy_2_PS_R1	1.52	0.80
E3_clay_2_PS_R1	1.65	0.91	E3_sandy_2_PS_R1	1.68	0.53
E1_clay_2_PS_R2	1.69	0.59	E1_sandy_2_PS_R2	1.70	0.92
E2_clay_2_PS_R2	1.73	0.52	E2_sandy_2_PS_R2	1.53	0.92
E3_clay_2_PS_R2	1.57	0.86	E3_sandy_2_PS_R2	1.52	0.64
E1_clay_2_PS_R3	1.69	0.95	E1_sandy_2_PS_R3	1.71	1.02
E2_clay_2_PS_R3	1.74	0.93	E2_sandy_2_PS_R3	1.63	0.74
E3_clay_2_PS_R3	1.57	1.00	E3_sandy_2_PS_R3	1.42	0.59
E1_clay_3_PS_R1	1.87	1.63	E1_sandy_3_PS_R1	1.65	1.17
E2_clay_3_PS_R1	1.21	0.23	E2_sandy_3_PS_R1	1.70	0.94
E3_clay_3_PS_R1	1.31	0.49	E3_sandy_3_PS_R1	1.61	1.00
E1_clay_3_PS_R2	1.68	1.06	E1_sandy_3_PS_R2	1.70	1.05
E2_clay_3_PS_R2	1.47	0.27	E2_sandy_3_PS_R2	1.68	0.77
E3_clay_3_PS_R2	1.13	0.16	E3_sandy_3_PS_R2	1.60	0.84
E1_clay_3_PS_R3	1.89	1.70	E1_sandy_3_PS_R3	1.67	1.02
E2_clay_3_PS_R3	1.43	0.25	E2_sandy_3_PS_R3	1.66	0.99
E3_clay_3_PS_R3	1.31	0.28	E3_sandy_3_PS_R3	1.56	0.72

B

Clay			Sandy		
**Samples**	**260/280**	**260/230**	**Samples**	**260/280**	**260/230**
E1_clay_1_FS_R1	1.78	0.42	E1_sandy_1_FS_R1	1.52	0.92
E2_clay_1_FS_R1	1.50	0.22	E2_sandy_1_FS_R1	1.51	0.76
E3_clay_1_FS_R1	1.44	0.12	E3_sandy_1_FS_R1	1.44	0.64
E1_clay_1_FS_R2	1.83	0.47	E1_sandy_1_FS_R2	1.49	0.69
E2_clay_1_FS_R2	1.65	0.26	E2_sandy_1_FS_R2	1.55	0.79
E3_clay_1_FS_R2	1.48	0.11	E3_sandy_1_FS_R2	1.39	0.61
E1_clay_1_FS_R3	1.82	0.48	E1_sandy_1_FS_R3	1.57	0.72
E2_clay_1_FS_R3	1.59	0.22	E2_sandy_1_FS_R3	1.43	0.60
E3_clay_1_FS_R3	1.52	0.15	E3_sandy_1_FS_R3	1.42	0.60
E1_clay_2_FS_R1	1.78	0.18	E1_sandy_2_FS_R1	1.54	0.67
E2_clay_2_FS_R1	1.76	0.20	E2_sandy_2_FS_R1	1.33	0.52
E3_clay_2_FS_R1	1.57	0.10	E3_sandy_2_FS_R1	1.44	0.38
E1_clay_2_FS_R2	1.82	0.31	E1_sandy_2_FS_R2	1.37	0.61
E2_clay_2_FS_R2	1.80	0.08	E2_sandy_2_FS_R2	1.36	0.63
E3_clay_2_FS_R2	1.59	0.16	E3_sandy_2_FS_R2	1.19	0.58
E1_clay_2_FS_R3	1.85	0.39	E1_sandy_2_FS_R3	1.33	0.73
E2_clay_2_FS_R3	1.76	0.19	E2_sandy_2_FS_R3	1.35	0.66
E3_clay_2_FS_R3	1.56	0.13	E3_sandy_2_FS_R3	1.31	0.63
E1_clay_3_FS_R1	1.85	0.57	E1_sandy_3_FS_R1	1.30	0.58
E2_clay_3_FS_R1	1.78	0.32	E2_sandy_3_FS_R1	1.27	0.65
E3_clay_3_FS_R1	1.53	0.29	E3_sandy_3_FS_R1	1.23	0.49
E1_clay_3_FS_R2	1.86	0.63	E1_sandy_3_FS_R2	1.33	0.57
E2_clay_3_FS_R2	1.74	0.57	E2_sandy_3_FS_R2	1.39	0.56
E3_clay_3_FS_R2	1.47	0.56	E3_sandy_3_FS_R2	1.38	0.61
E1_clay_3_FS_R3	1.85	0.73	E1_sandy_3_FS_R3	1.39	0.63
E2_clay_3_FS_R3	1.74	0.43	E2_sandy_3_FS_R3	1.13	0.53
E3_clay_3_FS_R3	1.55	0.43	E3_sandy_3_FS_R3	1.36	0.55

References

Kuramae, E.E., Yergeau, E., Wong, L.C., Pijl, A.S., van Veen, J.A., Kowalchuk, G.A., 2012. Soil characteristics more strongly influence soil bacterial communities than land-use type. FEMS Microbiol Ecol 79, 12-24.
